# Changes in inflammatory factors in the Brown Norway rat model of food allergy

**DOI:** 10.1186/s12865-021-00398-9

**Published:** 2021-01-26

**Authors:** Qingling Zhu, Junli Wang, Jingqiu Ma, Xiaoyang Sheng, Feng Li

**Affiliations:** 1 Department of Child and Adolescent Healthcare, MOE-Shanghai Key Laboratory of Children’s Environmental Health, Xinhua Hospital Affiliated to Shanghai Jiao Tong University School of Medicine, No. 1665 Kongjiang Road, Yangpu Shanghai, 200092 China; 2 Department of Children Healthcare, Quanzhou Women’s and Children’s Hospital, Quanzhou, 362000 Fujian China

**Keywords:** S100A8/A9, Food allergy, Immunoglobulin E, Intestinal inflammation, Animal experimentation

## Abstract

**Background:**

The role of serum S100A8/A9 in intestinal inflammation has been confirmed, and its role in food allergy is currently being investigated.

**Objective:**

To explore the levels of S100A8/A9 and inflammatory factors, including Toll-like receptors 4 (TLR4), Nuclear transcription factors (NF-κB) and Tumor necrosis factor α (TNF-α), in mild food allergies.

**Methods:**

Eighty 3-week-old male Brown Norway rats were used. Forty rats were randomly assigned to the ovalbumin-sensitized experimental group, while 40 rats were assigned to the normal saline sham-sensitized control group. Body weight and length and the levels of serum ovalbumin-specific IgE (OVA-IgE), histamine, Th1-associated and Th2-associated factors, S100A8/A9 and inflammation-associated cytokines were compared.

**Results:**

Through the evaluation of OVA-IgE level and Th1/Th2 balance in the experimental group, a successful IgE-mediated food allergy model was constructed. Compared with the control group, the experimental group had higher serum S100A8/A9 levels on days 21, 28, 35 and 42 (all *P <* 0.05); higher TLR4 levels on days 28, 35 and 42 (all *P <* 0.05); higher TNF-α levels on days 28, 35 and 42 (all *P <* 0.05); higher NF-κB levels on days 35 and 42 (all *P* < 0.05); and higher IL-1β and IL-6 levels on days 7 to 42 (all *P <* 0.05). Moreover, positive correlations were found between the serum levels of S100A8/A9 and inflammation-associated cytokines [TNF-α: *r* = 0.378, *P* = 0.039; IL-1β: *r* = 0.679, *P* = 0.000; IL-6: *r* = 0.590, *P* = 0.001].

**Conclusion:**

S100A8/A9 and inflammatory-related factors, including TLR4, NF-κB, TNF-α, IL-6 and IL-1β, is closely related to food allergies. Moreover, immune and inflammatory factors interact with each other in food allergies, which may provide insight into food allergy causes and treatments.

**Supplementary Information:**

The online version contains supplementary material available at 10.1186/s12865-021-00398-9.

## Background

Food allergy is a major health problem among infants and young children worldwide [1]. The prevalence of food allergies decreases with increasing age, but they reduce the quality of life in both the individuals who suffer from them and their family members [2].

Calprotectin, also known S100A8/A9, is widely distributed in human cells, tissues and body fluids, and is a type of calcium and zinc binding protein that belongs to the S100 family. It’s the main protein in eosinophils and macrophages and an important part in the innate immune system [3, 4], which involved in the regulation of cellular processes, such as cell cycle progression and differentiation, and has been identified as a biomarker for acute and chronic inflammation and cardiac failure [3, 5]. In addition, S100A8/A9 has been identified as an important endogenous damage-related molecule that binds to TLR4 and plays a key role in the process of inflammatory amplification; as such, it is a promising new therapeutic target [6]. Toll-like receptors (TLR) play a vital role in the activation of innate immunity, which protects the intestinal epithelial barrier, provides tolerance, and promotes healing [7]. The activation of TLRs leads to the upregulation of major histocompatibility complex molecules and costimulatory factors, the expression of pro-inflammatory cytokines, and the activation of adaptive immunity [8]. Besides, the maturation of dendritic cells requires signal mediation via TLRs, especially TLR4 [9]. Furthermore, S100A8/A9 is both an endogenous ligand and a direct exogenous ligand of TLR4 [10].

Allergy is a state of inflammation [11]. The aim of this study was to investigate the expression of plasma S100A8/A9 in IgE-mediated food allergies and its correlation with immune- and inflammatory-related factors, such as TLR4, nuclear transcription factors (NF-κB) and tumor necrosis factor α (TNF-α), terleukin-6 (IL-6) and IL-1β, to provide more experimental data regarding the mechanisms underlying food allergies.

## Results

### Body lengths and weights of allergic rats were not lower than those of the control rats

In this study, no accidental death or loss of an animal occurred in either the experimental group or the control group. The baseline body weights and body lengths in the experimental group and control group were the same (*P* > 0.05), ensuring a comparable baseline.

There were no differences in body length or weight between the experimental group and the control group (all *P* > 0.05), which was also observed in our previous study [12] (Table 1).

**Table 1:** The baseline of control group and experimental group (*N* = 30)

	Weight ($\overline{}x$ ± s, g)	Length ($\overline{}x$ ± s, cm)
Control group	99.71 ± 3.57	27.76 ± 0.46
Experimental group	93.62 ± 3.16	28.98 ± 1.68
*Z* value	−1.16	−0.55
*P* value	0.246	0.582

### Serum levels of OVA-specific IgE and histamine were increased in sensitized rats

Antigen-specific IgE and histamine are vitally important mediators of food allergies. Thus, the ovalbumin (OVA)-specific IgE and histamine levels in the serum of rats were assessed. As shown in our previous study, significantly higher levels of OVA-specific IgE and histamine were detected in the serum of rats treated with OVA compared with those treated with normal saline [13].

**Fig. 1:**
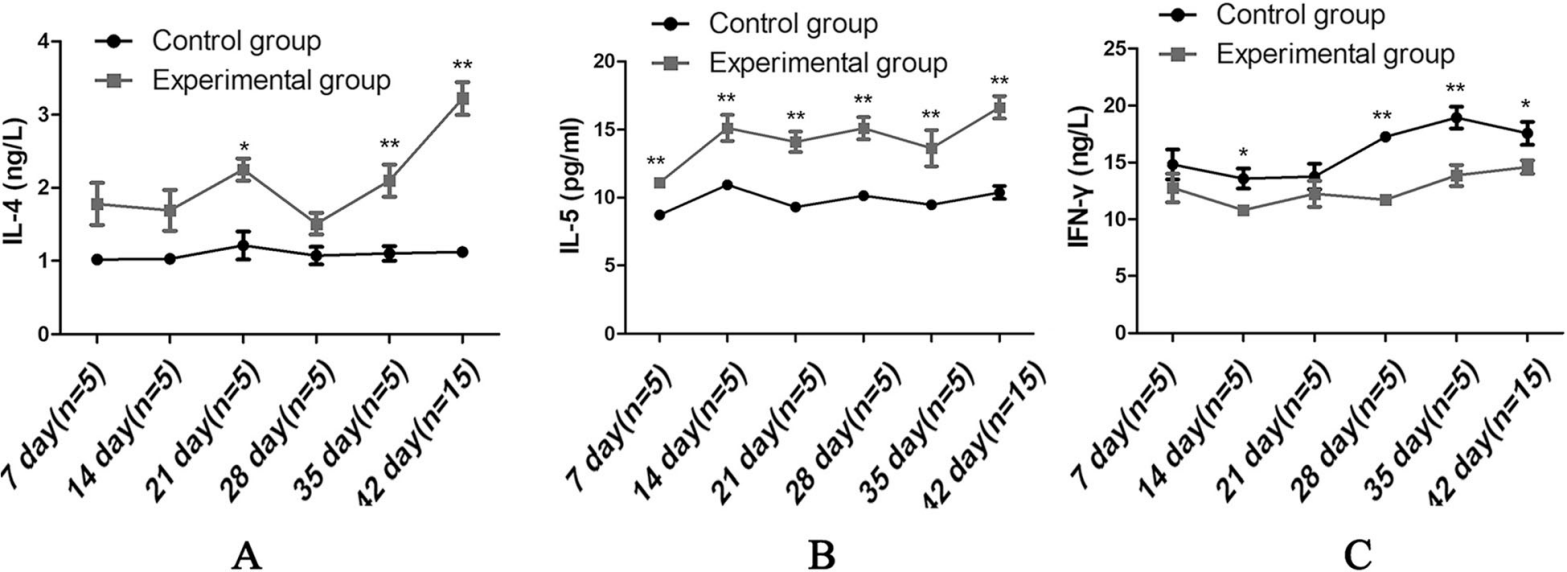
Changes in Th2-associated cytokines and Th1-associated cytokines. The mesenteric lymph nodes were collected weekly after sensitization and the final challenge in both the allergic and control groups. **a**, **b** The levels of the Th2-associated cytokines IL-4 and IL-5 in the mesenteric lymph nodes. **c** The level of the Th1-associated cytokine IFN-γ in the mesenteric lymph nodes. All levels were detected by ELISA every 7 days from day 7 to day 35 (*n* = 5/group) and on day 42 (*n* = 15/group). Data are represented as the means ± SEM. *P<0.05; **P<0.01

The expression levels of the Th2-associated cytokines IL-4 and IL-5 were obviously increased in the experimental group compared to those in control group. The level of IL-4 between the two groups was statistically differences on days 21, 35 and 42; and the level of IL-5 between the two groups was statistically differences on days 7, 14, 21, 28, 35 and 42 (all *P*<0.05). In addition, the expression levels of the Th1-associated cytokine interferon (IFN)-γ were lower in the experimental group than that in control group, and there were statistically differences on days 14, 28, 35 and 42 (all *P*<0.05). As seen in Fig. 1.

### Level of S100A8/A9 in the plasma and levels of the inflammation-associated cytokines TLR4, TNF-α, NF-κB, IL-1β and IL-6

**Fig. 2:**
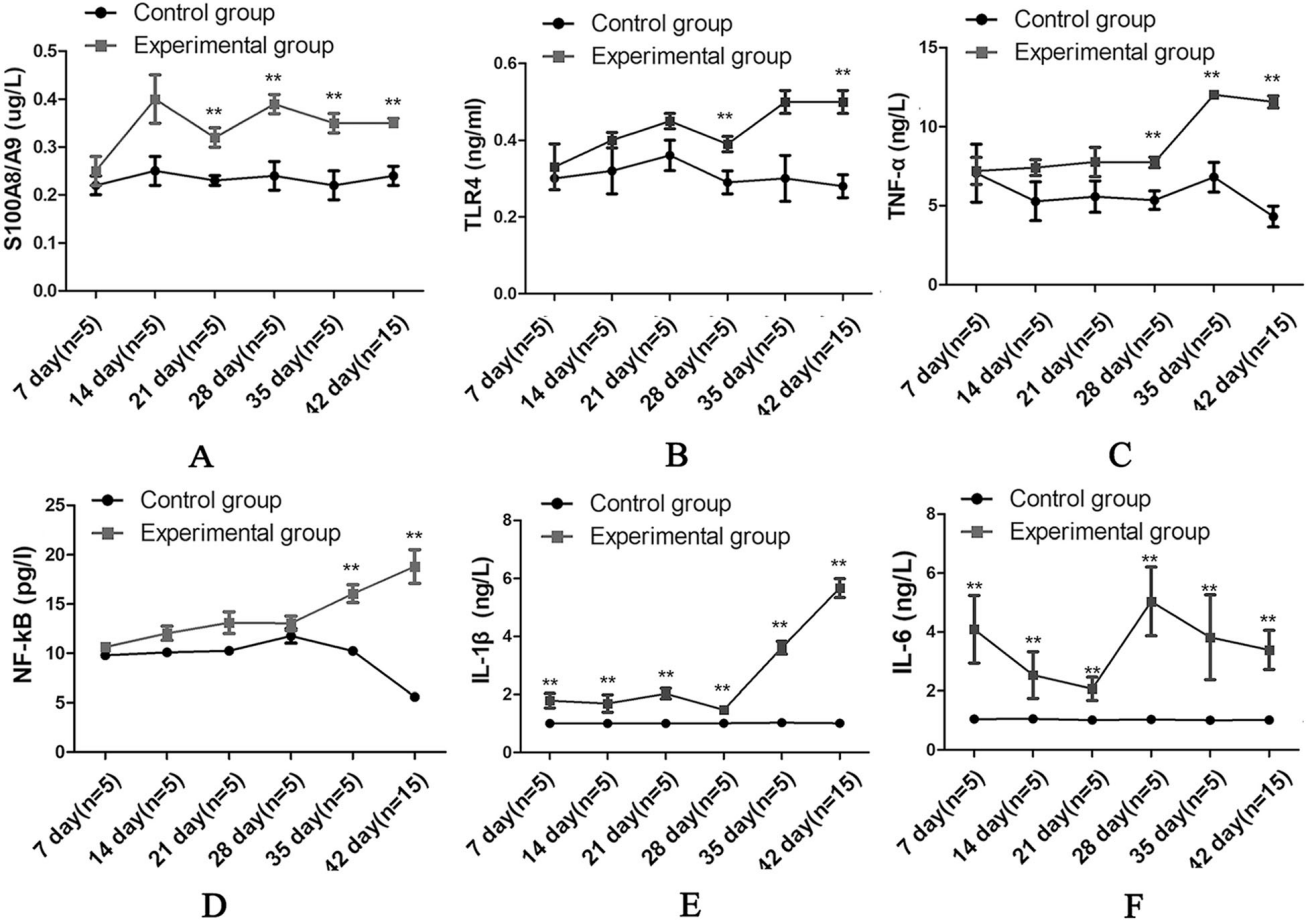
The levels of S100A8/A9 in the plasma and the levels of the inflammation-associated cytokines. ELISA was used to detect the levels of S100A8/A9, TLR4, TNF-α, NF-κB, IL-1β and IL-6. **a** The levels of S100A8/A9 in the plasma. **b** The levels of TLR4; **c** TNF-α; **d** NF-κB; **e** IL-1β; and **f** IL-6. *P<0.05; **P<0.01

**Fig. 3:**
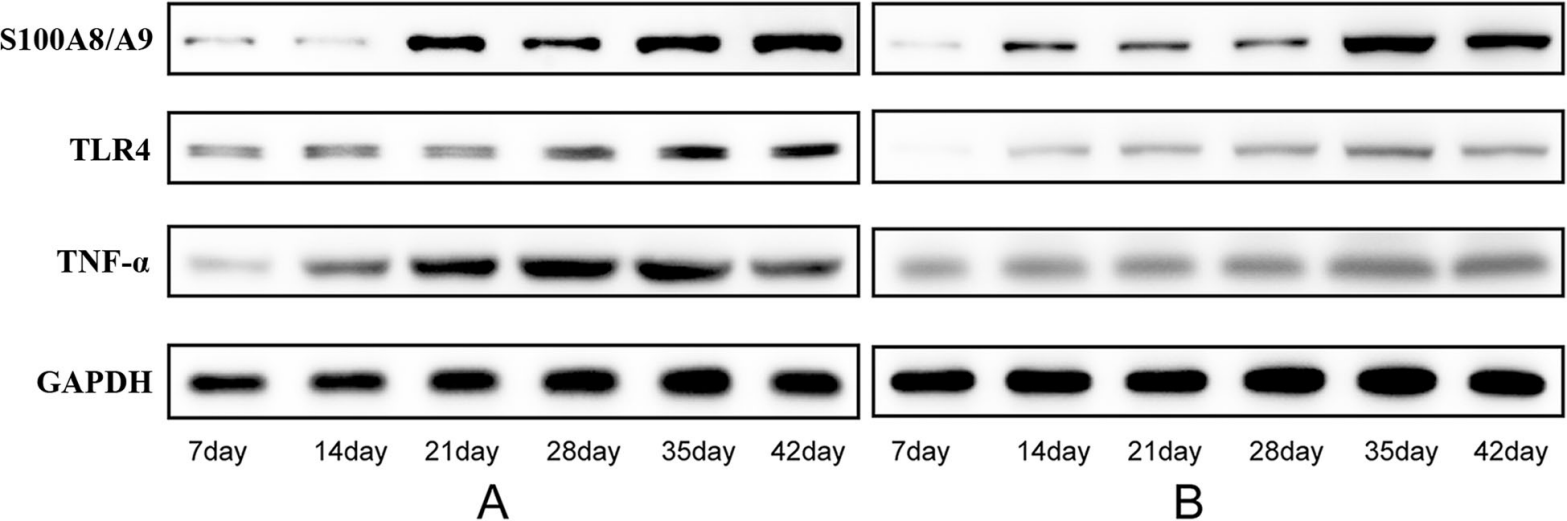
The levels of S100A8/A9, TLR4 and TNF-α. Weston blot was used to detect the levels of S100A8/A9, TLR4 and TNF-α. **a** S100A8/A9, TLR4 and TNF-α levels in the experimental group; **b** S100A8/A9, TLR4 and TNF-α levels in the control group

The plasma levels of S100A8/A9 were increased in the experimental group than that in control group, especially on days 21, 28, 35 and 42 (all *P*<0.05), as seen in Fig. 2(a) and Fig. 3(a and b).

The levels of TLR4 in the mesenteric lymph nodes in the experimental group were higher than those in the control group, and significant differences were found on days 28, 35 and 42 (all *P*<0.05), as seen in Fig. 2(b) and Fig. 3(a and b).

The levels of TNF-α in the mesenteric lymph nodes in the experimental group were higher than those in the control group, and significant differences were found on days 28, 35 and 42 (all *P*<0.05), as seen in Fig. 2(c) and Fig. 3(a and b).

The levels of NF-κB in the mesenteric lymph nodes in the experimental group were higher than those in the control group, and significant differences were found on days 35 and 42 (both *P*<0.05), as seen in Fig. 2(d).

The levels of IL-1β and IL-6 in the mesenteric lymph nodes in the experimental group were higher than those in the control group, and significant differences were found from day 7 to day 42 (all *P*<0.05), as seen in Fig. 2 (e and f).

### Correlation of the level of S100A8/A9 with the levels of TLR4, TNF-α, NF-κB, IL-1β and IL-6

The level of S100A8/A9 was positively correlated with the levels of TNF-α, IL-1β and IL-6 *(r* = 0.378, *P* = 0.039; *r* = 0.679, *P* = 0.000; *r* = 0.590, *P* = 0.001).

In addition, there were also some correlations between the levels of inflammatory factors. For example, the level of TNF-α was positively correlated with the levels of TLR4, IL-1β and IL-6 (*r* = 0.914, *P* = 0.000; *r* = 0.633, *P* = 0.000; *r* = 0.552, *P* = 0.002). There was a positive correlation between the level of TLR4 and the levels of IL-1β and IL-6 but not TNF-α (*r* = 0.588, *P* = 0.001; *r* = 0.534, *P* = 0.002).

## Discussion

Brown Norway (BN) rats were more suitable for IgE-mediated food allergy animal model research [13, 14]. What’s more, BN rats showed the same pattern of protein recognition as humans [15]. In addition, the allergic animal model using BN rats was suitable for the dynamic analysis of serum-specific antibodies and that allergic processes and symptoms similar to those observed in human allergies can be produced by the oral administration of allergenic drugs without the use of immune adjuvants [16]. Our results showed that the administration of OVA, an antigen, could lead to elevated levels of antigen-specific IgE in the serum and inflammation in the intestine of the rats. Besides, an obvious Th1/Th2 imbalance in this model was observed.

In our study, Th2-related cytokines such as IL-4 and IL-5 were upregulated in the experimental group, whereas the level of the Th1-related cytokine IFN-γ in the experimental group was lower/normal compared to that in the control group. In addition, the levels of OVA-IgE and histamine were higher in the experimental group. These results suggested the occurrence of allergies. Our study found that the S100A8/A9 level in the experimental group was markedly higher. Plasma S100A8/A9 acts as an endogenous ligand for TLR4. An elevated level of S100A8/A9 is not a prerequisite for inflammation but is associated with immunoregulatory factors induced by allergy-related factors. In addition, the level of TLR4 was also higher in the experimental group. S100A8/A9 is known to be involved in binding TLR4 and activating TLR4-Myd88 signalling [17]. TLR4 plays a pivotal role in controlling allergic inflammation [17], which was found to be significantly increased in Food allergies patients but undetectable in many patients with treated food allergies [18]. The activation of TLR4-dependent signals inhibits allergic responses to food antigens in experimental animals, and TLR4 knockout mice are highly susceptible to developing food allergies [19]. The induction of tolerance has been shown to be associated with the suppression of TLR expression [20]. TLRs are induced or expressed by a variety of cell types in the gastrointestinal tract, including intestinal epithelial cells, intestinal macrophages and dendritic cells [21], and CD4, CD25, and Treg cells. It has been reported that the levels of TLR4 are significantly elevated in intestinal epithelial cells and macrophages in active inflammatory bowel disease [22]. In addition, the TLR4 pathway has been shown to play a key role in allergic inflammation [23]. In our research, a positive correlation was found between the levels of S100A8/A9 and TLR4, which was in line with the results mentioned above. Similarly, Huang et al. noted that an increased serum level of S100A8/A9 can be used as a potential biomarker of trichloroethylene-induced hypersensitivity dermatitis for clinical diagnosis and drug treatment [24]. It was also observed that the stimulation of S100A8/A9 could induce an increase in the levels of pro-inflammatory cytokines (IL-6 and TNF-α) [25], which was confirmed in our study.

S100A8/A9 has been found to play a vital role in inhibiting acute inflammation by regulating the activity of pro-inflammatory cytokines in liver injury, cardiac injury, and arthritis, also confirmed in our OVA allergic model. TNF-α, a key pro-inflammatory cytokine, plays a central role in the pathogenesis of chronic immune-mediated diseases [26]. In our study, the levels of pro-inflammatory cytokines, including TNF-α, NF-κB, IL-1β and IL-6, were clearly higher in allergic rats, which agree with the findings of the study mentioned above [27]. S100A8/A9 plays an important role in the pathogenic mechanism by activating macrophages through the activation of TLR4-dependent signalling cascades. This result indicated that there is an inflammation-related reaction dominated by S100A8A9 via TLR4-dependent signalling in allergies, although the exact mechanism of this reaction is still unclear. Moreover, the finding that the level of S100A8/A9 is positively correlated with the levels of TNF-α, IL-1β and IL-6 further supports that hypothesis.

Transcription factors are key molecules involved in the determination of the Th1/Th2 balance. For example, NF-κB is actively involved in the Th1/Th2 balance, specifically in the class switching to IgE (9). NF-κB is helpful in the activation of the co-stimulatory molecules CD40 and CD154. In our study, the level of NF-κB in allergic rats supported its role in food allergies to some extent.

In our study, the allergic rats showed mild symptoms, providing an ideal animal model for studying the effects of persistent mild allergic symptoms on growth and development. Besides, the roles of serum S100A8/A9 and inflammatory-related factors in food allergies and possible underlying mechanisms were preliminary explored, which provided more basic data and laboratory evidence regarding food allergies.

Inevitably, there were some limitations to this study. First, the most important limitation was the lack of species diversity. Although animal models are similar to human conditions to a certain extent, they still cannot fully represent a series of pathological changes in humans. Thus, the results of these models are limited. The role of S100A8/A9 in the development of human food allergies remains to be investigated in further research. Second, to avoid the effect of sex on the results, only male BN rats were selected; however, this results in a lack of knowledge of these processes in female BN rats, leading inevitably to bias. Finally, this study only initially explored the expression level of serum S100A8/A9 and its relationship with the levels of other inflammatory and immune-related factors. Further research is needed to understand the role of S100A8/A9 in food allergies.

## Materials and methods

### Animals

Eighty 3-week-old male specific pathogen free (SPF) grade BN rats, obtained from the Animal Center of the Chinese Academy of Medical Sciences (Shanghai, China), were maintained on an OVA-free rodent diet under specific pathogen-free conditions in accordance with standard guidelines for the care and use of animals [10]. All of the rats received adaptive feeding for 4 days with a 12-h light-dark cycle at a constant temperature (23 ± 3) °C and a relative humidity of 50 ~ 70%.

Eighty male BN rats were divided into an allergic group and a control group by a random number table. During the experiment, the body length and weight of each rat were monitored every 7 days (7 ~ 42 days). Every 7 days after receiving the intervention with OVA (experimental group) or normal saline (control group), 5 rats (days 7, 14, 21, 28, 35) or 15 rats (day 42) in each group were sacrificed by cervical dislocation by a trained opperator after low-dose anesthetics and were and were buried properly in *Experimental animal burial area*.

The primary outcome included the levels of S100A8/A9 and levels of IL-4, IL-5, IFN-γ, TLR4, NF-κB, TNF-α, IL-6 and IL-1β in the mesenteric lymph nodes. The secondary outcome the levels of OVA-specific IgE and histamine in the blood, the expression of S100A8/A9, TLR4, NF-kB and TNF-α by Western blot and the correlations of S100A8A9 with TLR4, NF-κB, TNF-α, IL-6 and IL-1β.

### Sensitization and challenge

Rats in the experimental group underwent oral gavage with 1 mg of OVA (1 mg ml − 1 per rat) (Sigma-V, purity > 95%) daily for 41 days. On day 42, each rat in the experimental group was orally challenged with 100 mg of OVA. Meanwhile, rats in the control group were given an equal volume of NS. The details were described previously [12]. All rats were sacrificed by cervical dislocation. After euthanasia, blood and tissue samples and mesenteric lymph nodes were collected for further analysis. The observers and statisticians were blinded to the group assignment.

### Enzyme-linked immunosorbent assay

The levels of S100A8/A9, OVA-specific IgE and histamine in the blood and levels of IL-4, IL-5, IFN-γ, TLR4, NF-κB, TNF-α, IL-6 and IL-1β in the mesenteric lymph nodes were determined by ELISA with commercial reagent kits (sbjbio, Inc., Nanjing, Jiangsu, China) according to the manufacturer’s instructions; the specific methods were described in detail in our previous study [12].

### Western blot analysis

Total proteins were extracted from the mesenteric lymph nodes with RIPA buffer supplemented with a cocktail of protease inhibitors, including PMSF. An equal amount of 20 μg of protein was fully electrophoresed on 10% SDS polyacrylamide gels and transferred to polyvinylidene fluoride membranes. After blocking with 5% skim milk at room temperature for 1 h, the membranes were incubated with primary antibodies overnight at 4 °C. The primary antibodies against GAPDH were obtained from Abcam and were diluted at a 1:10000 ratio, and the other antibodies were as follows: anti-TLR4 (1:1000, NOVUS), anti-S100A8/A9 (1:1000, NOVUS), anti-NF-kB p65 (0.5 μg/ml, Abcam) and anti-TNF-α (1:1000, Abcam). Subsequently, the membranes were incubated with goat anti-rabbit or anti-mouse secondary antibodies (diluted at a 1:5000 ratio), and then an ECL Detection System (Millipore) was used to detect the protein.

### Statistical analyses

Statistical analysis was performed using the SPSS software package (version 16.0 for Windows; SPSS, Inc., Chicago, IL, USA). All values are shown as the means ± S.E. Student’s t tests and Mann-Whitney U tests were used for the comparisons of different groups. A simple regression analysis was carried out to estimate the correlations of S100A8A9 with TLR4, NF-κB, TNF-α, IL-6 and IL-1β, and the significance of those correlations was determined with Spearman’s correlation tests. All of the tests were 2-sided, and a probability (*P*-value) < 0.05 indicated significance.

## Supplementary Information

**Additional file 1: S1.** The levels of S100A8/A9 in the experimental group. **S2**. The levels of S100A8/A9 in the control group. **S3**. The levels of TLR4 in the experimental group. **S4**. The levels of TLR4 in the control group. **S5**. The levels of TNF-α in the experimental group. **S6**. The levels of TNF-α in the control group. **S7**. The levels of GAPDH in the experimental group. **S**8. The levels of GAPDH in the control group.

## Abbreviations

TLR: Toll-like receptors
NF: Nuclear transcription factors
TNF-α: Tumor necrosis factor α
IL: Interleukin
OVA: Ovalbumin
IFN: Interferon
BN: Brown Norway
SPF: Specific pathogen free
