# Supplementary material for: Changes in inflammatory factors in the Brown Norway rat model of food allergy
Source: BMC Immunol. 2021 Jan 26;22:8. doi: 10.1186/s12865-021-00398-9 (PMC7839196; doi:10.1186/s12865-021-00398-9)
Supplement: Supplementary file 1 — Additional file 1: S1. The levels of S100A8/A9 in the experimental group. S2. The levels of S100A8/A9 in the control group. S3. The levels of TLR4 in the experimental group. S4. The levels of TLR4 in the control group. S5. The levels of TNF-α in the experimental group. S6. The levels of TNF-α in the control group. S7. The levels of GAPDH in the experimental group. S8. The levels of GAPDH in the control group. [file 12865_2021_398_MOESM1_ESM.pdf]

Manuscript Title: Changes in inflammatory factors in the Brown Norway rat model of food allergy

Qingling Zhu, PhD [a,b], Junli Wang, PhD [a], Jingqiu Ma, PhD [a], Xiaoyang Sheng, PhD [a]*, Feng Li, PhD [a]*

[a] Department of Child and Adolescent Healthcare, MOE-Shanghai Key Laboratory of Children's Environmental Health, Xinhua Hospital Affiliated to Shanghai Jiao Tong University School of Medicine, Shanghai, China 200092 ;

[b] Department of Children Healthcare, Quanzhou Women's and Children's Hospital, Quanzhou, Fujian, China 362000

***Corresponding author**:

1. Feng Li, Department of Child and Adolescent Healthcare, Xinhua Hospital Affiliated to Shanghai Jiao Tong University School of Medicine, No. 1665 Kongjiang Road, Yangpu Shanghai, China, 200092

Tel: +86- 13512111965，E-mail: lifeng@xinhuamed.com.cn

2. Xiaoyang Sheng, Department of Child and Adolescent Healthcare, Xinhua Hospital Affiliated to Shanghai Jiao Tong University School of Medicine, No. 1665 Kongjiang Road, Yangpu Shanghai, China, 200092

Tel: +86-13818072645，E-mail: shengxiaoyangcn@aliyun.com

Supplementary Info File:

Multiple exposures of Weston blot in Fig 3

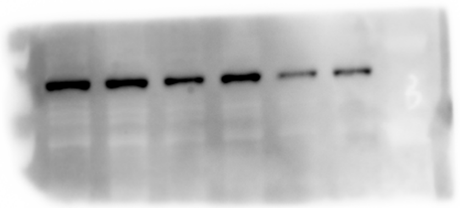

Sup 1 The levels of S100A8/A9 in the experimental group

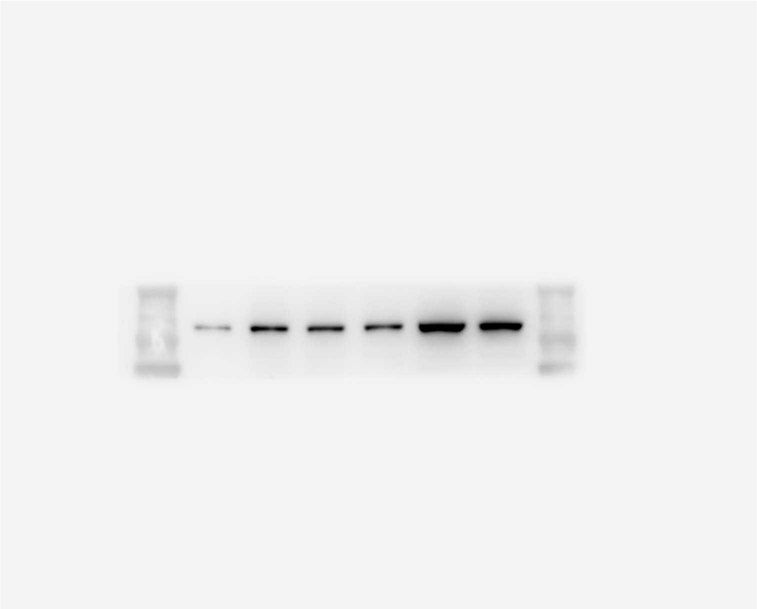

Sup 2 The levels of S100A8/A9 in the control group

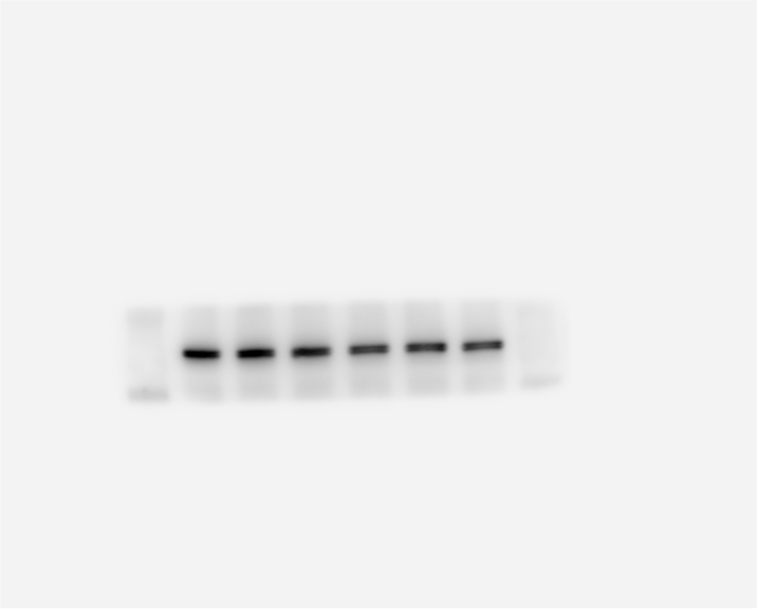

Sup 3 The levels of TLR4 in the experimental group

Sup 4 The levels of TLR4 in the control group

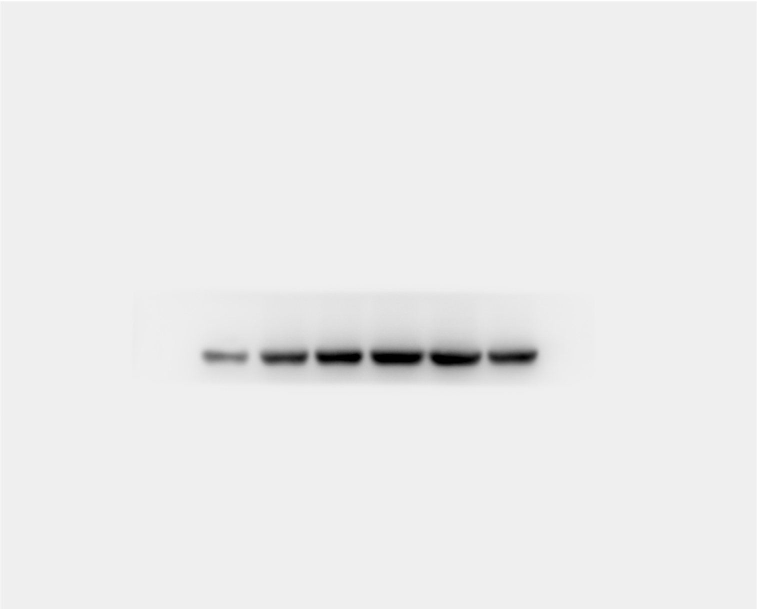

Sup 5 The levels of TNF-α in the experimental group

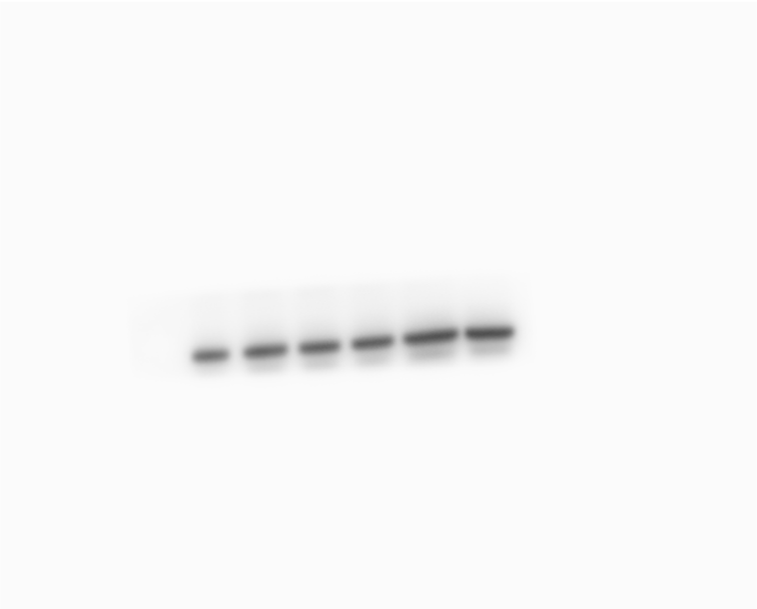

Sup 6 The levels of TNF-α in the control group

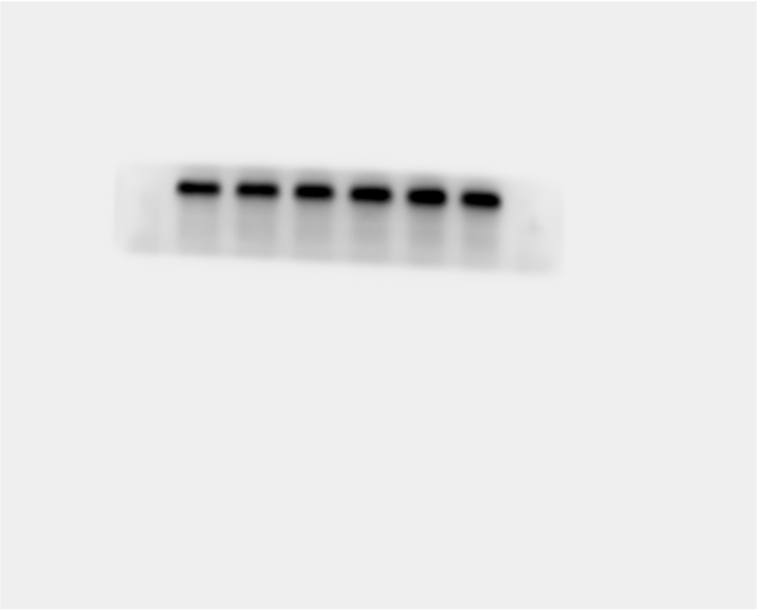

Sup 7 The levels of GAPDH in the experimental group

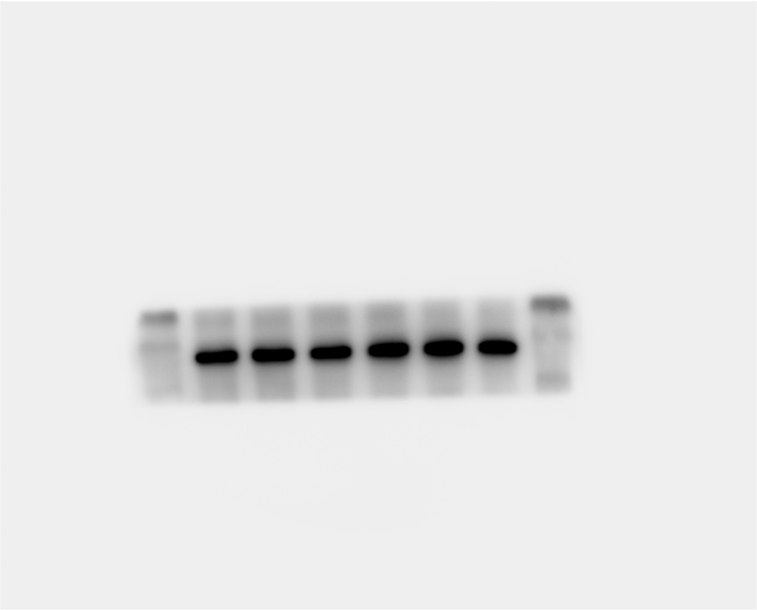

Sup 8 The levels of GAPDH in the control group
